# 1′-Methyl-2-oxo-5′-phenyl­spiro­[indoline-3,3′-pyrrolidine]-4′,4′-dicarbo­nitrile

**DOI:** 10.1107/S1600536809053094

**Published:** 2009-12-16

**Authors:** Mohammad Reza Nabid, Seyed Jamal Tabatabaei Rezaei, Yousef Fazaeli, Seik Weng Ng

**Affiliations:** a Department of Chemistry, General Campus, Shahid Beheshti University, Tehran 1983963113, Iran; b Department of Chemistry, University of Malaya, 50603 Kuala Lumpur, Malaysia

## Abstract

The title spiro-compound, C_20_H_16_N_4_O, crystallizes with four independent mol­ecules in the asymmetric unit. In all of them, the oxindole unit is planar, the r.m.s. deviations ranging from 0.07 to 0.08 Å, while the pyrrolinyl ring adopts an envelope conformation (with the N atom representing the flap). In the crystal, adjacent mol­ecules are linked by N—H⋯N and N—H⋯O hydrogen bonds.

## Related literature

For the synthesis, see: Ghandi *et al.* (2009 ▶).
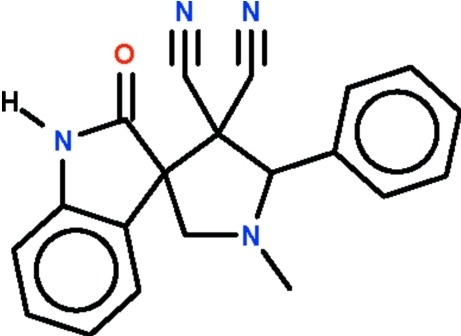


## Experimental

### 

#### Crystal data

C_20_H_16_N_4_O
*M* *_r_* = 328.37
Monoclinic, 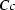

*a* = 14.3750 (3) Å
*b* = 15.8411 (3) Å
*c* = 30.7348 (5) Å
β = 101.888 (1)°
*V* = 6848.7 (2) Å^3^
*Z* = 16
Mo *K*α radiation
μ = 0.08 mm^−1^
*T* = 295 K
0.20 × 0.15 × 0.05 mm

#### Data collection

Bruker SMART APEX diffractometer
39181 measured reflections
7860 independent reflections
4866 reflections with *I* > 2σ(*I*)
*R*_int_ = 0.062

#### Refinement

*R*[*F* ^2^ > 2σ(*F* ^2^)] = 0.072
*wR*(*F* ^2^) = 0.198
*S* = 1.03
7860 reflections
901 parameters
2 restraints
H-atom parameters constrained
Δρ_max_ = 0.57 e Å^−3^
Δρ_min_ = −0.25 e Å^−3^

### 

Data collection: *APEX2* (Bruker, 2009 ▶); cell refinement: *SAINT* (Bruker, 2009 ▶); data reduction: *SAINT*; program(s) used to solve structure: *SHELXS97* (Sheldrick, 2008 ▶); program(s) used to refine structure: *SHELXL97* (Sheldrick, 2008 ▶); molecular graphics: *X-SEED* (Barbour, 2001 ▶); software used to prepare material for publication: *publCIF* (Westrip, 2009 ▶).

## Supplementary Material

Crystal structure: contains datablocks global, I. DOI: 10.1107/S1600536809053094/bt5137sup1.cif

Structure factors: contains datablocks I. DOI: 10.1107/S1600536809053094/bt5137Isup2.hkl

Additional supplementary materials:  crystallographic information; 3D view; checkCIF report

## Figures and Tables

**Table 1:** Hydrogen-bond geometry (Å, °)

*D*—H⋯*A*	*D*—H	H⋯*A*	*D*⋯*A*	*D*—H⋯*A*
N1—H1⋯O2	0.86	2.08	2.867 (6)	153
N5—H5⋯N16	0.86	2.40	3.184 (8)	151
N9—H9⋯O4^i^	0.86	2.10	2.874 (6)	150
N13—H13⋯N8	0.86	2.33	3.120 (8)	152

Symmetry code: (i) 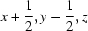.

**Fig. 1.**
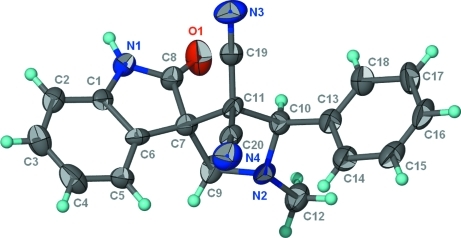
Anisotropic displacement ellipsoid plot (Barbour, 2001) of one of the four independent molecules of C20H16N4O; ellipsoids are drawn at the 50% probability level and H atoms of arbitrary radius.

## supplementary crystallographic information

### Experimental

Sarcosine (89 mg,1.0 mmol), benzaldehyde (104 mg, 1.0 mmol) and 2-oxo-(3*H*)-indol-3-ylidine-malononitrile (195 mg, 1.0 mmol) in dry toluene (30 ml) containing molecular sieves (500 mg,3 Å) were heated for five hours. The solvent was removed under reduced pressure and the residue was subjected to column chromatography on silica gel by using hexane–ethyl acetate (9:1) as eluent to give the product. This was recrystallized from methanol.

### Refinement

Due to the absence of anomalous scatterers, Friedel pairs were merged. Hydrogen atoms were placed in calculated positions (C—H 0.93–0.98, N–H 0.86 Å) and were included in the refinement in the riding model approximation, with *U*(H) set to 1.2–1.5*U*(C, N).

### Crystal data

**Table:**

C_20_H_16_N_4_O	*F*(000) = 2752
*M**_r_* = 328.37	*D*_x_ = 1.274 Mg m^−^^3^
Monoclinic, *C**c*	Mo *K*α radiation, λ = 0.71073 Å
Hall symbol: C -2yc	Cell parameters from 8592 reflections
*a* = 14.3750 (3) Å	θ = 2.1–28.2°
*b* = 15.8411 (3) Å	µ = 0.08 mm^−^^1^
*c* = 30.7348 (5) Å	*T* = 295 K
β = 101.888 (1)°	Block, colorless
*V* = 6848.7 (2) Å^3^	0.20 × 0.15 × 0.05 mm
*Z* = 16	

### Data collection

**Table:**

Bruker SMART APEX diffractometer	4866 reflections with *I* > 2σ(*I*)
Radiation source: fine-focus sealed tube	*R*_int_ = 0.062
graphite	θ_max_ = 27.5°, θ_min_ = 1.4°
ω scans	*h* = −18→18
39181 measured reflections	*k* = −19→20
7860 independent reflections	*l* = −39→39

### Refinement

**Table:**

Refinement on *F*^2^	Primary atom site location: structure-invariant direct methods
Least-squares matrix: full	Secondary atom site location: difference Fourier map
*R*[*F*^2^ > 2σ(*F*^2^)] = 0.072	Hydrogen site location: inferred from neighbouring sites
*wR*(*F*^2^) = 0.198	H-atom parameters constrained
*S* = 1.03	*w* = 1/[σ^2^(*F*_o_^2^) + (0.1236*P*)^2^] where *P* = (*F*_o_^2^ + 2*F*_c_^2^)/3
7860 reflections	(Δ/σ)_max_ = 0.001
901 parameters	Δρ_max_ = 0.57 e Å^−^^3^
2 restraints	Δρ_min_ = −0.25 e Å^−^^3^

### Fractional atomic coordinates and isotropic or equivalent isotropic displacement parameters (Å^2^)

**Table:**

	*x*	*y*	*z*	*U*_iso_*/*U*_eq_	
O1	0.5000 (4)	0.4227 (3)	0.50000 (15)	0.0745 (13)	
O2	0.4094 (3)	0.6148 (2)	0.39678 (14)	0.0600 (11)	
O3	0.4609 (4)	0.3302 (3)	0.23104 (15)	0.0803 (15)	
O4	0.0523 (3)	0.6404 (2)	0.33482 (15)	0.0636 (11)	
N1	0.4163 (4)	0.4444 (3)	0.42872 (18)	0.0458 (12)	
H1	0.4154	0.4987	0.4286	0.055*	
N2	0.4967 (4)	0.1845 (3)	0.49913 (18)	0.0488 (13)	
N3	0.2501 (4)	0.3842 (4)	0.5061 (2)	0.0745 (16)	
N4	0.2459 (4)	0.1583 (3)	0.4414 (2)	0.0672 (17)	
N5	0.3614 (3)	0.7448 (3)	0.36747 (17)	0.0372 (11)	
H5	0.3350	0.7298	0.3409	0.045*	
N6	0.5214 (3)	0.6988 (3)	0.51471 (15)	0.0430 (11)	
N7	0.3036 (5)	0.8377 (4)	0.5185 (2)	0.082 (2)	
N8	0.2140 (4)	0.6247 (4)	0.43970 (19)	0.0704 (16)	
N9	0.5469 (4)	0.3105 (3)	0.30183 (16)	0.0463 (12)	
H9	0.5508	0.2564	0.3018	0.056*	
N10	0.4660 (3)	0.5696 (3)	0.23200 (18)	0.0523 (13)	
N11	0.7072 (5)	0.3702 (4)	0.2223 (2)	0.0810 (18)	
N12	0.7171 (4)	0.5954 (4)	0.2875 (2)	0.0672 (17)	
N13	0.1014 (4)	0.5112 (3)	0.36387 (18)	0.0450 (12)	
H13	0.1294	0.5269	0.3901	0.054*	
N14	−0.0612 (3)	0.5524 (3)	0.21707 (16)	0.0424 (12)	
N15	0.1564 (5)	0.4170 (4)	0.2118 (2)	0.0767 (19)	
N16	0.2441 (4)	0.6315 (4)	0.28951 (18)	0.0688 (16)	
C1	0.3771 (3)	0.3941 (3)	0.39179 (17)	0.0370 (11)	
C2	0.3392 (4)	0.4208 (4)	0.3492 (2)	0.0464 (15)	
H2	0.3324	0.4778	0.3422	0.056*	
C3	0.3114 (4)	0.3591 (4)	0.3173 (2)	0.0578 (15)	
H3	0.2843	0.3747	0.2883	0.069*	
C4	0.3235 (5)	0.2750 (4)	0.3280 (2)	0.0640 (18)	
H4	0.3053	0.2347	0.3059	0.077*	
C5	0.3618 (4)	0.2492 (4)	0.3707 (2)	0.0449 (15)	
H5A	0.3705	0.1922	0.3774	0.054*	
C6	0.3872 (4)	0.3099 (3)	0.4034 (2)	0.0390 (12)	
C7	0.4306 (4)	0.3034 (3)	0.45186 (18)	0.0409 (12)	
C8	0.4556 (4)	0.3972 (3)	0.4645 (2)	0.0442 (14)	
C9	0.5172 (4)	0.2412 (4)	0.4647 (2)	0.0542 (17)	
H9A	0.5254	0.2091	0.4389	0.065*	
H9B	0.5752	0.2726	0.4758	0.065*	
C10	0.4364 (4)	0.2321 (3)	0.52346 (18)	0.0444 (12)	
H10	0.4738	0.2781	0.5398	0.053*	
C11	0.3635 (4)	0.2699 (3)	0.48389 (18)	0.0411 (12)	
C12	0.5809 (5)	0.1476 (5)	0.5260 (3)	0.070 (2)	
H12A	0.5633	0.1121	0.5483	0.105*	
H12B	0.6223	0.1917	0.5401	0.105*	
H12C	0.6132	0.1145	0.5076	0.105*	
C13	0.3948 (4)	0.1791 (4)	0.5558 (2)	0.0459 (13)	
C14	0.3623 (5)	0.0975 (4)	0.5459 (2)	0.0583 (17)	
H14	0.3668	0.0731	0.5189	0.070*	
C15	0.3227 (5)	0.0519 (4)	0.5767 (3)	0.0604 (18)	
H15	0.2993	−0.0023	0.5701	0.072*	
C16	0.3192 (6)	0.0888 (5)	0.6169 (3)	0.074 (2)	
H16	0.2931	0.0585	0.6374	0.088*	
C17	0.3521 (5)	0.1673 (5)	0.6275 (2)	0.067 (2)	
H17	0.3496	0.1902	0.6550	0.081*	
C18	0.3904 (5)	0.2143 (4)	0.5967 (2)	0.0583 (16)	
H18	0.4127	0.2688	0.6036	0.070*	
C19	0.3028 (4)	0.3354 (4)	0.4968 (2)	0.0456 (13)	
C20	0.2986 (4)	0.2049 (3)	0.4606 (2)	0.0438 (13)	
C21	0.3701 (4)	0.8286 (3)	0.38201 (19)	0.0398 (12)	
C22	0.3460 (5)	0.9015 (4)	0.3568 (2)	0.0491 (16)	
H22	0.3179	0.8992	0.3267	0.059*	
C23	0.3664 (4)	0.9775 (4)	0.3790 (3)	0.0619 (17)	
H23	0.3509	1.0276	0.3634	0.074*	
C24	0.4092 (5)	0.9816 (4)	0.4239 (3)	0.0642 (18)	
H24	0.4218	1.0337	0.4378	0.077*	
C25	0.4328 (5)	0.9084 (4)	0.4476 (3)	0.0574 (18)	
H25	0.4641	0.9109	0.4772	0.069*	
C26	0.4098 (3)	0.8304 (3)	0.42720 (19)	0.0360 (11)	
C27	0.4273 (3)	0.7417 (3)	0.44390 (16)	0.0390 (11)	
C28	0.3992 (4)	0.6902 (3)	0.40008 (17)	0.0391 (11)	
C29	0.5275 (4)	0.7205 (4)	0.4685 (2)	0.0445 (14)	
H29A	0.5524	0.6731	0.4545	0.053*	
H29B	0.5693	0.7685	0.4683	0.053*	
C30	0.4324 (3)	0.6537 (3)	0.51070 (16)	0.0384 (11)	
H30	0.4379	0.5992	0.4963	0.046*	
C31	0.3634 (3)	0.7113 (3)	0.47756 (16)	0.0388 (11)	
C32	0.6039 (5)	0.6535 (5)	0.5385 (2)	0.0609 (18)	
H32A	0.5956	0.6404	0.5679	0.091*	
H32B	0.6596	0.6878	0.5403	0.091*	
H32C	0.6113	0.6021	0.5230	0.091*	
C33	0.3986 (4)	0.6381 (4)	0.55392 (18)	0.0401 (13)	
C34	0.3450 (5)	0.5668 (4)	0.5557 (2)	0.0597 (18)	
H34	0.3347	0.5284	0.5323	0.072*	
C35	0.3062 (6)	0.5526 (6)	0.5931 (3)	0.081 (3)	
H35	0.2666	0.5066	0.5944	0.097*	
C36	0.3282 (6)	0.6091 (6)	0.6286 (3)	0.076 (2)	
H36	0.3058	0.5982	0.6543	0.091*	
C37	0.3791 (6)	0.6767 (5)	0.6269 (2)	0.073 (2)	
H37	0.3908	0.7139	0.6508	0.087*	
C38	0.4162 (5)	0.6931 (4)	0.5890 (2)	0.0545 (16)	
H38	0.4526	0.7413	0.5877	0.065*	
C39	0.3304 (4)	0.7836 (4)	0.50075 (18)	0.0462 (13)	
C40	0.2798 (4)	0.6639 (4)	0.4556 (2)	0.0482 (15)	
C41	0.5833 (3)	0.3617 (3)	0.33911 (17)	0.0387 (11)	
C42	0.6212 (5)	0.3355 (4)	0.3813 (2)	0.0476 (15)	
H42	0.6285	0.2785	0.3883	0.057*	
C43	0.6487 (4)	0.3979 (4)	0.4138 (2)	0.0595 (16)	
H43	0.6752	0.3822	0.4428	0.071*	
C44	0.6370 (4)	0.4812 (4)	0.4031 (2)	0.0628 (17)	
H44	0.6532	0.5214	0.4255	0.075*	
C45	0.6009 (5)	0.5082 (4)	0.3590 (2)	0.0545 (18)	
H45	0.5955	0.5653	0.3517	0.065*	
C46	0.5736 (4)	0.4454 (3)	0.32667 (19)	0.0369 (11)	
C47	0.5280 (4)	0.4517 (3)	0.27773 (18)	0.0412 (12)	
C48	0.5057 (4)	0.3566 (4)	0.2666 (2)	0.0473 (15)	
C49	0.4408 (5)	0.5089 (4)	0.2629 (3)	0.0576 (18)	
H49A	0.3862	0.4758	0.2486	0.069*	
H49B	0.4251	0.5376	0.2884	0.069*	
C50	0.5240 (4)	0.5239 (3)	0.20686 (18)	0.0444 (12)	
H50	0.4858	0.4787	0.1902	0.053*	
C51	0.5967 (3)	0.4840 (3)	0.24662 (18)	0.0407 (12)	
C52	0.3795 (5)	0.6080 (5)	0.2036 (3)	0.068 (2)	
H52A	0.3438	0.6372	0.2221	0.102*	
H52B	0.3409	0.5643	0.1874	0.102*	
H52C	0.3983	0.6471	0.1832	0.102*	
C53	0.5665 (4)	0.5768 (4)	0.1750 (2)	0.0469 (15)	
C54	0.5981 (5)	0.6575 (4)	0.1841 (2)	0.0525 (16)	
H54	0.5917	0.6826	0.2107	0.063*	
C55	0.6387 (6)	0.7021 (5)	0.1550 (3)	0.074 (2)	
H55	0.6586	0.7571	0.1622	0.089*	
C56	0.6509 (6)	0.6690 (6)	0.1164 (3)	0.077 (3)	
H56	0.6798	0.6999	0.0971	0.093*	
C57	0.6195 (6)	0.5884 (7)	0.1065 (3)	0.084 (3)	
H57	0.6277	0.5641	0.0799	0.101*	
C58	0.5758 (5)	0.5417 (5)	0.1349 (2)	0.0617 (18)	
H58	0.5532	0.4877	0.1271	0.074*	
C59	0.6572 (4)	0.4177 (4)	0.2320 (3)	0.0554 (16)	
C60	0.6622 (4)	0.5478 (4)	0.2700 (2)	0.0445 (13)	
C61	0.0914 (3)	0.4266 (3)	0.34933 (19)	0.0401 (12)	
C62	0.1147 (5)	0.3531 (4)	0.3747 (2)	0.0506 (17)	
H62	0.1421	0.3556	0.4048	0.061*	
C63	0.0954 (5)	0.2774 (4)	0.3531 (3)	0.0630 (17)	
H63	0.1118	0.2274	0.3687	0.076*	
C64	0.0519 (6)	0.2739 (4)	0.3086 (3)	0.065 (2)	
H64	0.0370	0.2215	0.2953	0.078*	
C65	0.0302 (5)	0.3457 (4)	0.2836 (2)	0.0541 (17)	
H65	0.0025	0.3428	0.2535	0.065*	
C66	0.0512 (4)	0.4239 (3)	0.30506 (19)	0.0400 (12)	
C67	0.0322 (3)	0.5126 (3)	0.28778 (16)	0.0373 (11)	
C68	0.0611 (4)	0.5636 (3)	0.33100 (17)	0.0413 (11)	
C69	−0.0690 (4)	0.5321 (4)	0.2623 (2)	0.0540 (17)	
H69A	−0.1100	0.4836	0.2625	0.065*	
H69B	−0.0953	0.5795	0.2757	0.065*	
C70	0.0253 (3)	0.6005 (3)	0.22002 (16)	0.0382 (11)	
H70	0.0187	0.6551	0.2342	0.046*	
C71	0.0952 (3)	0.5429 (3)	0.25311 (16)	0.0380 (11)	
C72	−0.1442 (4)	0.6002 (4)	0.1939 (2)	0.0535 (16)	
H72A	−0.2006	0.5671	0.1927	0.080*	
H72B	−0.1370	0.6129	0.1643	0.080*	
H72C	−0.1492	0.6518	0.2097	0.080*	
C73	0.0560 (4)	0.6148 (3)	0.17656 (18)	0.0387 (12)	
C74	0.1120 (4)	0.6823 (4)	0.1719 (2)	0.0543 (16)	
H74	0.1281	0.7210	0.1950	0.065*	
C75	0.1452 (5)	0.6936 (5)	0.1331 (3)	0.070 (2)	
H75	0.1830	0.7403	0.1307	0.084*	
C76	0.1245 (6)	0.6395 (6)	0.0989 (3)	0.078 (2)	
H76	0.1499	0.6466	0.0736	0.093*	
C77	0.0632 (6)	0.5718 (5)	0.1024 (2)	0.0692 (19)	
H77	0.0446	0.5353	0.0785	0.083*	
C78	0.0305 (5)	0.5589 (4)	0.1408 (2)	0.0576 (16)	
H78	−0.0085	0.5130	0.1431	0.069*	
C79	0.1282 (4)	0.4718 (4)	0.2300 (2)	0.0527 (15)	
C80	0.1817 (4)	0.5926 (4)	0.27405 (19)	0.0483 (14)	

### Atomic displacement parameters (Å^2^)

**Table:**

	*U*^11^	*U*^22^	*U*^33^	*U*^12^	*U*^13^	*U*^23^
O1	0.087 (3)	0.075 (3)	0.055 (3)	−0.038 (3)	0.000 (2)	−0.001 (2)
O2	0.090 (3)	0.034 (2)	0.061 (3)	0.007 (2)	0.027 (2)	−0.0034 (18)
O3	0.106 (4)	0.077 (3)	0.050 (2)	−0.044 (3)	−0.003 (2)	0.000 (2)
O4	0.098 (3)	0.033 (2)	0.066 (3)	0.004 (2)	0.032 (2)	0.0004 (18)
N1	0.056 (3)	0.030 (2)	0.054 (3)	−0.013 (2)	0.017 (2)	0.001 (2)
N2	0.054 (3)	0.035 (3)	0.067 (3)	0.011 (2)	0.034 (3)	0.017 (2)
N3	0.067 (3)	0.058 (3)	0.108 (5)	0.015 (3)	0.040 (3)	0.000 (3)
N4	0.073 (4)	0.046 (3)	0.086 (5)	−0.019 (3)	0.022 (3)	−0.001 (3)
N5	0.042 (3)	0.035 (2)	0.036 (3)	−0.006 (2)	0.012 (2)	0.003 (2)
N6	0.031 (2)	0.054 (3)	0.043 (3)	0.0048 (19)	0.0055 (19)	0.010 (2)
N7	0.099 (5)	0.078 (4)	0.076 (4)	0.030 (4)	0.032 (4)	0.001 (3)
N8	0.052 (3)	0.104 (5)	0.052 (3)	−0.011 (3)	0.004 (3)	−0.010 (3)
N9	0.059 (3)	0.030 (2)	0.051 (3)	−0.002 (2)	0.014 (2)	−0.006 (2)
N10	0.042 (3)	0.057 (3)	0.062 (3)	0.014 (2)	0.021 (2)	0.028 (3)
N11	0.078 (4)	0.050 (3)	0.124 (5)	0.003 (3)	0.043 (4)	−0.011 (3)
N12	0.062 (4)	0.057 (3)	0.087 (5)	−0.003 (3)	0.025 (3)	−0.004 (3)
N13	0.057 (3)	0.036 (3)	0.041 (3)	−0.004 (2)	0.009 (2)	−0.003 (2)
N14	0.039 (3)	0.042 (2)	0.047 (3)	0.002 (2)	0.012 (2)	0.003 (2)
N15	0.093 (4)	0.069 (4)	0.078 (4)	0.039 (3)	0.040 (4)	0.006 (3)
N16	0.049 (3)	0.100 (5)	0.056 (3)	−0.017 (3)	0.009 (3)	0.003 (3)
C1	0.036 (2)	0.034 (3)	0.046 (3)	−0.006 (2)	0.021 (2)	0.003 (2)
C2	0.037 (3)	0.049 (4)	0.055 (4)	−0.002 (3)	0.014 (3)	0.011 (3)
C3	0.049 (3)	0.078 (5)	0.047 (3)	−0.016 (3)	0.012 (2)	0.003 (3)
C4	0.070 (4)	0.075 (5)	0.053 (4)	−0.032 (3)	0.027 (3)	−0.019 (3)
C5	0.052 (4)	0.033 (3)	0.057 (4)	−0.008 (3)	0.029 (3)	−0.002 (3)
C6	0.037 (3)	0.027 (3)	0.057 (4)	0.001 (2)	0.021 (3)	−0.004 (3)
C7	0.040 (3)	0.034 (3)	0.052 (3)	−0.005 (2)	0.018 (2)	0.003 (2)
C8	0.047 (3)	0.034 (3)	0.054 (4)	−0.010 (2)	0.017 (3)	0.004 (3)
C9	0.036 (3)	0.062 (4)	0.069 (4)	0.006 (3)	0.021 (3)	0.024 (3)
C10	0.041 (3)	0.046 (3)	0.049 (3)	0.000 (2)	0.017 (2)	0.009 (2)
C11	0.046 (3)	0.028 (3)	0.051 (3)	0.001 (2)	0.013 (2)	0.009 (2)
C12	0.064 (4)	0.074 (5)	0.080 (5)	0.023 (4)	0.033 (4)	0.030 (4)
C13	0.037 (3)	0.048 (3)	0.053 (4)	0.003 (3)	0.011 (2)	0.010 (3)
C14	0.057 (4)	0.057 (4)	0.067 (4)	0.013 (3)	0.027 (3)	0.015 (3)
C15	0.052 (4)	0.048 (4)	0.084 (5)	0.006 (3)	0.021 (4)	0.020 (4)
C16	0.068 (4)	0.074 (5)	0.092 (6)	0.030 (4)	0.049 (4)	0.051 (5)
C17	0.069 (4)	0.087 (5)	0.053 (4)	0.009 (4)	0.028 (3)	0.028 (4)
C18	0.057 (4)	0.063 (4)	0.054 (4)	−0.007 (3)	0.010 (3)	0.009 (3)
C19	0.048 (3)	0.039 (3)	0.055 (4)	−0.006 (3)	0.023 (3)	0.003 (3)
C20	0.041 (3)	0.034 (3)	0.059 (4)	−0.002 (2)	0.018 (3)	0.008 (3)
C21	0.038 (3)	0.038 (3)	0.048 (3)	−0.001 (2)	0.021 (2)	0.003 (2)
C22	0.042 (3)	0.052 (4)	0.057 (4)	0.006 (3)	0.017 (3)	0.015 (3)
C23	0.062 (4)	0.034 (3)	0.098 (5)	0.010 (3)	0.037 (4)	0.020 (3)
C24	0.077 (4)	0.035 (3)	0.088 (5)	−0.005 (3)	0.037 (4)	−0.009 (4)
C25	0.056 (4)	0.044 (4)	0.073 (5)	−0.011 (3)	0.015 (3)	−0.019 (3)
C26	0.033 (2)	0.031 (3)	0.047 (3)	0.002 (2)	0.015 (2)	−0.001 (2)
C27	0.040 (3)	0.043 (3)	0.037 (3)	0.005 (2)	0.015 (2)	0.002 (2)
C28	0.047 (3)	0.034 (3)	0.041 (3)	−0.001 (2)	0.018 (2)	0.001 (2)
C29	0.037 (3)	0.050 (4)	0.051 (4)	0.004 (2)	0.020 (3)	0.007 (3)
C30	0.038 (3)	0.039 (3)	0.037 (3)	0.002 (2)	0.005 (2)	0.000 (2)
C31	0.035 (2)	0.046 (3)	0.036 (3)	0.001 (2)	0.009 (2)	0.004 (2)
C32	0.041 (3)	0.076 (4)	0.062 (4)	0.020 (3)	0.003 (3)	0.012 (3)
C33	0.038 (3)	0.048 (3)	0.033 (3)	0.001 (3)	0.002 (2)	0.007 (3)
C34	0.072 (4)	0.046 (4)	0.057 (4)	−0.009 (3)	0.004 (3)	0.004 (3)
C35	0.064 (4)	0.086 (6)	0.088 (6)	−0.026 (4)	0.004 (4)	0.045 (5)
C36	0.079 (5)	0.100 (6)	0.054 (4)	0.021 (5)	0.025 (4)	0.027 (4)
C37	0.107 (6)	0.072 (5)	0.037 (4)	0.018 (4)	0.011 (4)	0.004 (3)
C38	0.070 (4)	0.050 (4)	0.039 (3)	−0.010 (3)	0.002 (3)	−0.002 (3)
C39	0.048 (3)	0.056 (4)	0.039 (3)	0.009 (3)	0.018 (2)	0.004 (3)
C40	0.037 (3)	0.067 (4)	0.043 (3)	0.002 (3)	0.013 (3)	0.008 (3)
C41	0.036 (2)	0.041 (3)	0.040 (3)	0.002 (2)	0.012 (2)	0.002 (2)
C42	0.046 (3)	0.044 (3)	0.054 (4)	−0.003 (3)	0.013 (3)	0.010 (3)
C43	0.053 (3)	0.079 (5)	0.048 (3)	−0.015 (3)	0.014 (3)	0.005 (3)
C44	0.072 (4)	0.069 (4)	0.052 (4)	−0.025 (3)	0.023 (3)	−0.022 (3)
C45	0.072 (5)	0.036 (3)	0.063 (5)	−0.012 (3)	0.030 (4)	−0.010 (3)
C46	0.042 (3)	0.026 (2)	0.048 (3)	−0.008 (2)	0.023 (2)	0.000 (2)
C47	0.038 (3)	0.039 (3)	0.049 (3)	0.004 (2)	0.015 (2)	0.015 (2)
C48	0.051 (3)	0.049 (3)	0.045 (3)	−0.016 (3)	0.017 (3)	−0.003 (3)
C49	0.048 (4)	0.058 (4)	0.073 (5)	0.007 (3)	0.026 (3)	0.022 (3)
C50	0.041 (3)	0.042 (3)	0.052 (3)	0.001 (2)	0.012 (2)	0.007 (2)
C51	0.037 (3)	0.038 (3)	0.050 (3)	−0.003 (2)	0.014 (2)	0.004 (2)
C52	0.049 (4)	0.079 (5)	0.078 (5)	0.021 (3)	0.019 (3)	0.040 (4)
C53	0.042 (3)	0.057 (4)	0.044 (3)	0.000 (3)	0.015 (2)	0.014 (3)
C54	0.061 (4)	0.041 (3)	0.059 (4)	0.009 (3)	0.017 (3)	0.011 (3)
C55	0.069 (5)	0.054 (4)	0.098 (7)	0.005 (3)	0.015 (4)	0.031 (4)
C56	0.054 (4)	0.101 (7)	0.077 (6)	0.006 (4)	0.016 (4)	0.056 (5)
C57	0.083 (5)	0.126 (8)	0.043 (4)	0.044 (6)	0.011 (4)	0.010 (4)
C58	0.070 (4)	0.073 (4)	0.042 (4)	0.005 (3)	0.011 (3)	0.002 (3)
C59	0.047 (3)	0.031 (3)	0.091 (5)	0.000 (3)	0.021 (3)	0.002 (3)
C60	0.046 (3)	0.039 (3)	0.053 (3)	0.005 (3)	0.021 (3)	0.008 (3)
C61	0.031 (2)	0.039 (3)	0.052 (3)	−0.001 (2)	0.011 (2)	0.002 (3)
C62	0.050 (3)	0.045 (4)	0.062 (4)	0.004 (3)	0.024 (3)	0.019 (3)
C63	0.067 (4)	0.043 (4)	0.087 (5)	0.012 (3)	0.035 (4)	0.018 (3)
C64	0.077 (4)	0.039 (4)	0.088 (6)	−0.006 (3)	0.037 (4)	−0.007 (4)
C65	0.068 (4)	0.043 (4)	0.060 (4)	−0.011 (3)	0.033 (3)	−0.015 (3)
C66	0.041 (3)	0.039 (3)	0.044 (3)	0.003 (2)	0.019 (2)	0.001 (2)
C67	0.035 (2)	0.037 (3)	0.042 (3)	0.004 (2)	0.011 (2)	0.002 (2)
C68	0.052 (3)	0.032 (3)	0.044 (3)	0.003 (2)	0.020 (2)	0.002 (2)
C69	0.039 (3)	0.075 (5)	0.050 (4)	0.007 (3)	0.013 (3)	0.018 (3)
C70	0.036 (2)	0.034 (3)	0.043 (3)	0.001 (2)	0.005 (2)	−0.003 (2)
C71	0.036 (3)	0.042 (3)	0.037 (3)	0.004 (2)	0.010 (2)	0.002 (2)
C72	0.036 (3)	0.062 (4)	0.060 (4)	0.006 (3)	0.004 (3)	0.012 (3)
C73	0.036 (3)	0.041 (3)	0.040 (3)	0.002 (2)	0.008 (2)	0.007 (2)
C74	0.045 (3)	0.061 (4)	0.053 (4)	0.002 (3)	0.001 (3)	0.002 (3)
C75	0.054 (4)	0.078 (5)	0.079 (6)	−0.006 (3)	0.017 (4)	0.032 (4)
C76	0.079 (5)	0.104 (6)	0.063 (5)	0.029 (5)	0.043 (4)	0.029 (4)
C77	0.096 (5)	0.073 (4)	0.046 (4)	0.004 (4)	0.033 (4)	−0.004 (3)
C78	0.078 (4)	0.051 (3)	0.045 (3)	−0.005 (3)	0.016 (3)	−0.004 (3)
C79	0.056 (4)	0.051 (4)	0.051 (3)	0.016 (3)	0.011 (3)	0.008 (3)
C80	0.041 (3)	0.068 (4)	0.037 (3)	−0.001 (3)	0.010 (3)	0.004 (3)

### Geometric parameters (Å, °)

**Table:**

O1—C8	1.215 (7)	C30—C31	1.561 (7)
O2—C28	1.209 (6)	C30—H30	0.9800
O3—C48	1.223 (7)	C31—C40	1.459 (8)
O4—C68	1.232 (6)	C31—C39	1.477 (8)
N1—C8	1.352 (8)	C32—H32A	0.9600
N1—C1	1.406 (7)	C32—H32B	0.9600
N1—H1	0.8600	C32—H32C	0.9600
N2—C12	1.441 (8)	C33—C38	1.370 (8)
N2—C9	1.464 (8)	C33—C34	1.375 (9)
N2—C10	1.465 (7)	C34—C35	1.396 (11)
N3—C19	1.160 (7)	C34—H34	0.9300
N4—C20	1.133 (7)	C35—C36	1.395 (13)
N5—C28	1.350 (7)	C35—H35	0.9300
N5—C21	1.399 (7)	C36—C37	1.304 (11)
N5—H5	0.8600	C36—H36	0.9300
N6—C30	1.449 (7)	C37—C38	1.400 (10)
N6—C32	1.450 (7)	C37—H37	0.9300
N6—C29	1.480 (8)	C38—H38	0.9300
N7—C39	1.125 (8)	C41—C42	1.364 (8)
N8—C40	1.153 (8)	C41—C46	1.379 (7)
N9—C48	1.338 (8)	C42—C43	1.401 (9)
N9—C41	1.413 (7)	C42—H42	0.9300
N9—H9	0.8600	C43—C44	1.361 (9)
N10—C50	1.443 (7)	C43—H43	0.9300
N10—C49	1.450 (8)	C44—C45	1.415 (10)
N10—C52	1.492 (8)	C44—H44	0.9300
N11—C59	1.121 (8)	C45—C46	1.405 (9)
N12—C60	1.142 (8)	C45—H45	0.9300
N13—C68	1.344 (7)	C46—C47	1.515 (8)
N13—C61	1.410 (7)	C47—C49	1.538 (8)
N13—H13	0.8600	C47—C48	1.563 (8)
N14—C70	1.445 (6)	C47—C51	1.594 (7)
N14—C69	1.452 (8)	C49—H49A	0.9700
N14—C72	1.467 (7)	C49—H49B	0.9700
N15—C79	1.152 (8)	C50—C53	1.510 (8)
N16—C80	1.111 (8)	C50—C51	1.568 (7)
C1—C2	1.376 (8)	C50—H50	0.9800
C1—C6	1.380 (7)	C51—C60	1.466 (8)
C2—C3	1.384 (9)	C51—C59	1.492 (9)
C2—H2	0.9300	C52—H52A	0.9600
C3—C4	1.374 (9)	C52—H52B	0.9600
C3—H3	0.9300	C52—H52C	0.9600
C4—C5	1.375 (9)	C53—C54	1.366 (8)
C4—H4	0.9300	C53—C58	1.384 (9)
C5—C6	1.384 (8)	C54—C55	1.362 (10)
C5—H5A	0.9300	C54—H54	0.9300
C6—C7	1.496 (8)	C55—C56	1.341 (13)
C7—C8	1.560 (8)	C55—H55	0.9300
C7—C9	1.572 (8)	C56—C57	1.369 (12)
C7—C11	1.604 (7)	C56—H56	0.9300
C9—H9A	0.9700	C57—C58	1.390 (12)
C9—H9B	0.9700	C57—H57	0.9300
C10—C13	1.515 (7)	C58—H58	0.9300
C10—C11	1.553 (7)	C61—C66	1.365 (8)
C10—H10	0.9800	C61—C62	1.403 (8)
C11—C19	1.461 (8)	C62—C63	1.371 (10)
C11—C20	1.472 (8)	C62—H62	0.9300
C12—H12A	0.9600	C63—C64	1.385 (10)
C12—H12B	0.9600	C63—H63	0.9300
C12—H12C	0.9600	C64—C65	1.372 (10)
C13—C14	1.387 (9)	C64—H64	0.9300
C13—C18	1.387 (9)	C65—C66	1.407 (8)
C14—C15	1.400 (9)	C65—H65	0.9300
C14—H14	0.9300	C66—C67	1.507 (7)
C15—C16	1.378 (11)	C67—C69	1.536 (7)
C15—H15	0.9300	C67—C68	1.537 (7)
C16—C17	1.346 (11)	C67—C71	1.607 (7)
C16—H16	0.9300	C69—H69A	0.9700
C17—C18	1.403 (9)	C69—H69B	0.9700
C17—H17	0.9300	C70—C73	1.508 (7)
C18—H18	0.9300	C70—C71	1.568 (7)
C21—C26	1.389 (8)	C70—H70	0.9800
C21—C22	1.395 (8)	C71—C79	1.461 (8)
C22—C23	1.385 (10)	C71—C80	1.500 (8)
C22—H22	0.9300	C72—H72A	0.9600
C23—C24	1.391 (10)	C72—H72B	0.9600
C23—H23	0.9300	C72—H72C	0.9600
C24—C25	1.375 (10)	C73—C74	1.363 (8)
C24—H24	0.9300	C73—C78	1.400 (8)
C25—C26	1.393 (8)	C74—C75	1.383 (10)
C25—H25	0.9300	C74—H74	0.9300
C26—C27	1.499 (7)	C75—C76	1.341 (12)
C27—C29	1.520 (8)	C75—H75	0.9300
C27—C28	1.555 (7)	C76—C77	1.408 (11)
C27—C31	1.593 (7)	C76—H76	0.9300
C29—H29A	0.9700	C77—C78	1.371 (9)
C29—H29B	0.9700	C77—H77	0.9300
C30—C33	1.525 (8)	C78—H78	0.9300
			
C8—N1—C1	111.9 (4)	C34—C35—H35	120.8
C8—N1—H1	124.0	C37—C36—C35	122.3 (8)
C1—N1—H1	124.0	C37—C36—H36	118.8
C12—N2—C9	113.2 (5)	C35—C36—H36	118.8
C12—N2—C10	115.5 (5)	C36—C37—C38	119.9 (7)
C9—N2—C10	106.3 (4)	C36—C37—H37	120.0
C28—N5—C21	112.0 (5)	C38—C37—H37	120.0
C28—N5—H5	124.0	C33—C38—C37	119.7 (7)
C21—N5—H5	124.0	C33—C38—H38	120.1
C30—N6—C32	114.5 (5)	C37—C38—H38	120.1
C30—N6—C29	105.0 (4)	N7—C39—C31	178.6 (7)
C32—N6—C29	113.2 (5)	N8—C40—C31	177.4 (7)
C48—N9—C41	111.8 (5)	C42—C41—C46	123.6 (5)
C48—N9—H9	124.1	C42—C41—N9	127.3 (5)
C41—N9—H9	124.1	C46—C41—N9	109.1 (5)
C50—N10—C49	105.6 (5)	C41—C42—C43	117.5 (6)
C50—N10—C52	113.3 (5)	C41—C42—H42	121.3
C49—N10—C52	111.2 (5)	C43—C42—H42	121.3
C68—N13—C61	110.5 (5)	C44—C43—C42	120.6 (6)
C68—N13—H13	124.8	C44—C43—H43	119.7
C61—N13—H13	124.8	C42—C43—H43	119.7
C70—N14—C69	107.0 (4)	C43—C44—C45	121.8 (6)
C70—N14—C72	111.2 (4)	C43—C44—H44	119.1
C69—N14—C72	111.2 (5)	C45—C44—H44	119.1
C2—C1—C6	122.8 (5)	C46—C45—C44	117.2 (6)
C2—C1—N1	127.5 (5)	C46—C45—H45	121.4
C6—C1—N1	109.6 (5)	C44—C45—H45	121.4
C1—C2—C3	117.2 (6)	C41—C46—C45	119.2 (6)
C1—C2—H2	121.4	C41—C46—C47	109.7 (5)
C3—C2—H2	121.4	C45—C46—C47	131.0 (5)
C4—C3—C2	120.7 (6)	C46—C47—C49	119.5 (5)
C4—C3—H3	119.7	C46—C47—C48	100.4 (4)
C2—C3—H3	119.7	C49—C47—C48	112.7 (5)
C3—C4—C5	121.5 (6)	C46—C47—C51	115.0 (4)
C3—C4—H4	119.2	C49—C47—C51	101.6 (4)
C5—C4—H4	119.2	C48—C47—C51	107.6 (5)
C4—C5—C6	118.7 (6)	O3—C48—N9	126.9 (6)
C4—C5—H5A	120.7	O3—C48—C47	124.9 (5)
C6—C5—H5A	120.7	N9—C48—C47	108.2 (5)
C1—C6—C5	119.1 (6)	N10—C49—C47	106.6 (5)
C1—C6—C7	108.9 (5)	N10—C49—H49A	110.4
C5—C6—C7	132.0 (5)	C47—C49—H49A	110.4
C6—C7—C8	102.0 (4)	N10—C49—H49B	110.4
C6—C7—C9	116.1 (5)	C47—C49—H49B	110.4
C8—C7—C9	113.9 (5)	H49A—C49—H49B	108.6
C6—C7—C11	117.0 (4)	N10—C50—C53	115.1 (4)
C8—C7—C11	107.4 (4)	N10—C50—C51	98.7 (4)
C9—C7—C11	100.6 (4)	C53—C50—C51	115.7 (4)
O1—C8—N1	127.0 (5)	N10—C50—H50	108.9
O1—C8—C7	126.2 (5)	C53—C50—H50	108.9
N1—C8—C7	106.8 (5)	C51—C50—H50	108.9
N2—C9—C7	107.4 (5)	C60—C51—C59	106.1 (4)
N2—C9—H9A	110.2	C60—C51—C50	111.2 (4)
C7—C9—H9A	110.2	C59—C51—C50	112.6 (5)
N2—C9—H9B	110.2	C60—C51—C47	110.0 (5)
C7—C9—H9B	110.2	C59—C51—C47	115.3 (4)
H9A—C9—H9B	108.5	C50—C51—C47	101.6 (4)
N2—C10—C13	113.9 (4)	N10—C52—H52A	109.5
N2—C10—C11	100.0 (4)	N10—C52—H52B	109.5
C13—C10—C11	115.7 (4)	H52A—C52—H52B	109.5
N2—C10—H10	109.0	N10—C52—H52C	109.5
C13—C10—H10	109.0	H52A—C52—H52C	109.5
C11—C10—H10	109.0	H52B—C52—H52C	109.5
C19—C11—C20	105.9 (4)	C54—C53—C58	118.0 (6)
C19—C11—C10	113.9 (5)	C54—C53—C50	123.3 (6)
C20—C11—C10	111.6 (4)	C58—C53—C50	118.7 (6)
C19—C11—C7	113.6 (4)	C55—C54—C53	121.3 (7)
C20—C11—C7	109.5 (5)	C55—C54—H54	119.3
C10—C11—C7	102.4 (4)	C53—C54—H54	119.3
N2—C12—H12A	109.5	C56—C55—C54	122.1 (8)
N2—C12—H12B	109.5	C56—C55—H55	119.0
H12A—C12—H12B	109.5	C54—C55—H55	119.0
N2—C12—H12C	109.5	C55—C56—C57	117.7 (7)
H12A—C12—H12C	109.5	C55—C56—H56	121.1
H12B—C12—H12C	109.5	C57—C56—H56	121.1
C14—C13—C18	119.8 (6)	C56—C57—C58	121.8 (8)
C14—C13—C10	122.2 (6)	C56—C57—H57	119.1
C18—C13—C10	118.0 (5)	C58—C57—H57	119.1
C13—C14—C15	119.9 (7)	C53—C58—C57	119.1 (7)
C13—C14—H14	120.1	C53—C58—H58	120.4
C15—C14—H14	120.1	C57—C58—H58	120.4
C16—C15—C14	118.9 (7)	N11—C59—C51	176.0 (7)
C16—C15—H15	120.6	N12—C60—C51	176.3 (6)
C14—C15—H15	120.6	C66—C61—C62	122.1 (5)
C17—C16—C15	122.1 (7)	C66—C61—N13	109.9 (5)
C17—C16—H16	119.0	C62—C61—N13	128.0 (6)
C15—C16—H16	119.0	C63—C62—C61	117.2 (6)
C16—C17—C18	119.7 (7)	C63—C62—H62	121.4
C16—C17—H17	120.1	C61—C62—H62	121.4
C18—C17—H17	120.1	C62—C63—C64	121.2 (6)
C13—C18—C17	119.6 (7)	C62—C63—H63	119.4
C13—C18—H18	120.2	C64—C63—H63	119.4
C17—C18—H18	120.2	C65—C64—C63	121.6 (6)
N3—C19—C11	175.9 (6)	C65—C64—H64	119.2
N4—C20—C11	176.2 (6)	C63—C64—H64	119.2
C26—C21—C22	122.9 (5)	C64—C65—C66	117.8 (7)
C26—C21—N5	109.3 (5)	C64—C65—H65	121.1
C22—C21—N5	127.8 (6)	C66—C65—H65	121.1
C23—C22—C21	116.2 (6)	C61—C66—C65	120.0 (5)
C23—C22—H22	121.9	C61—C66—C67	109.3 (5)
C21—C22—H22	121.9	C65—C66—C67	130.5 (5)
C22—C23—C24	122.3 (6)	C66—C67—C69	116.8 (5)
C22—C23—H23	118.8	C66—C67—C68	100.9 (4)
C24—C23—H23	118.8	C69—C67—C68	113.2 (5)
C25—C24—C23	119.8 (6)	C66—C67—C71	115.2 (4)
C25—C24—H24	120.1	C69—C67—C71	101.9 (4)
C23—C24—H24	120.1	C68—C67—C71	109.1 (4)
C24—C25—C26	120.0 (7)	O4—C68—N13	125.2 (5)
C24—C25—H25	120.0	O4—C68—C67	125.9 (5)
C26—C25—H25	120.0	N13—C68—C67	108.8 (4)
C21—C26—C25	118.6 (5)	N14—C69—C67	106.3 (5)
C21—C26—C27	109.2 (4)	N14—C69—H69A	110.5
C25—C26—C27	132.0 (6)	C67—C69—H69A	110.5
C26—C27—C29	116.9 (5)	N14—C69—H69B	110.5
C26—C27—C28	101.6 (4)	C67—C69—H69B	110.5
C29—C27—C28	111.4 (4)	H69A—C69—H69B	108.7
C26—C27—C31	115.3 (4)	N14—C70—C73	115.4 (4)
C29—C27—C31	102.9 (4)	N14—C70—C71	99.3 (4)
C28—C27—C31	108.8 (4)	C73—C70—C71	113.4 (4)
O2—C28—N5	127.4 (5)	N14—C70—H70	109.5
O2—C28—C27	125.2 (5)	C73—C70—H70	109.5
N5—C28—C27	107.4 (4)	C71—C70—H70	109.5
N6—C29—C27	106.9 (4)	C79—C71—C80	106.9 (5)
N6—C29—H29A	110.3	C79—C71—C70	111.3 (4)
C27—C29—H29A	110.3	C80—C71—C70	109.6 (4)
N6—C29—H29B	110.3	C79—C71—C67	112.1 (4)
C27—C29—H29B	110.3	C80—C71—C67	113.7 (4)
H29A—C29—H29B	108.6	C70—C71—C67	103.3 (4)
N6—C30—C33	116.1 (4)	N14—C72—H72A	109.5
N6—C30—C31	101.4 (4)	N14—C72—H72B	109.5
C33—C30—C31	112.9 (4)	H72A—C72—H72B	109.5
N6—C30—H30	108.7	N14—C72—H72C	109.5
C33—C30—H30	108.7	H72A—C72—H72C	109.5
C31—C30—H30	108.7	H72B—C72—H72C	109.5
C40—C31—C39	107.8 (4)	C74—C73—C78	118.5 (6)
C40—C31—C30	110.6 (5)	C74—C73—C70	119.9 (5)
C39—C31—C30	111.3 (4)	C78—C73—C70	121.6 (5)
C40—C31—C27	112.7 (4)	C73—C74—C75	120.6 (7)
C39—C31—C27	111.4 (4)	C73—C74—H74	119.7
C30—C31—C27	103.1 (4)	C75—C74—H74	119.7
N6—C32—H32A	109.5	C76—C75—C74	122.2 (7)
N6—C32—H32B	109.5	C76—C75—H75	118.9
H32A—C32—H32B	109.5	C74—C75—H75	118.9
N6—C32—H32C	109.5	C75—C76—C77	117.8 (7)
H32A—C32—H32C	109.5	C75—C76—H76	121.1
H32B—C32—H32C	109.5	C77—C76—H76	121.1
C38—C33—C34	120.4 (6)	C78—C77—C76	120.7 (7)
C38—C33—C30	122.9 (5)	C78—C77—H77	119.6
C34—C33—C30	116.7 (5)	C76—C77—H77	119.6
C33—C34—C35	119.1 (7)	C77—C78—C73	120.0 (6)
C33—C34—H34	120.4	C77—C78—H78	120.0
C35—C34—H34	120.4	C73—C78—H78	120.0
C36—C35—C34	118.4 (7)	N15—C79—C71	178.2 (7)
C36—C35—H35	120.8	N16—C80—C71	178.0 (7)
			
C8—N1—C1—C2	−173.9 (6)	C48—N9—C41—C42	173.2 (6)
C8—N1—C1—C6	2.2 (7)	C48—N9—C41—C46	−5.9 (7)
C6—C1—C2—C3	−0.6 (8)	C46—C41—C42—C43	2.1 (9)
N1—C1—C2—C3	175.0 (5)	N9—C41—C42—C43	−176.8 (5)
C1—C2—C3—C4	−1.2 (9)	C41—C42—C43—C44	0.4 (9)
C2—C3—C4—C5	1.1 (9)	C42—C43—C44—C45	−2.8 (9)
C3—C4—C5—C6	1.0 (9)	C43—C44—C45—C46	2.6 (10)
C2—C1—C6—C5	2.7 (8)	C42—C41—C46—C45	−2.1 (8)
N1—C1—C6—C5	−173.7 (5)	N9—C41—C46—C45	176.9 (5)
C2—C1—C6—C7	180.0 (5)	C42—C41—C46—C47	−179.3 (5)
N1—C1—C6—C7	3.6 (6)	N9—C41—C46—C47	−0.3 (6)
C4—C5—C6—C1	−2.8 (8)	C44—C45—C46—C41	−0.2 (9)
C4—C5—C6—C7	−179.3 (6)	C44—C45—C46—C47	176.3 (6)
C1—C6—C7—C8	−7.1 (5)	C41—C46—C47—C49	128.8 (5)
C5—C6—C7—C8	169.7 (6)	C45—C46—C47—C49	−48.0 (8)
C1—C6—C7—C9	−131.5 (5)	C41—C46—C47—C48	5.1 (5)
C5—C6—C7—C9	45.3 (8)	C45—C46—C47—C48	−171.6 (6)
C1—C6—C7—C11	109.8 (5)	C41—C46—C47—C51	−110.0 (5)
C5—C6—C7—C11	−73.4 (8)	C45—C46—C47—C51	73.2 (7)
C1—N1—C8—O1	174.8 (6)	C41—N9—C48—O3	−173.2 (6)
C1—N1—C8—C7	−6.7 (7)	C41—N9—C48—C47	9.2 (7)
C6—C7—C8—O1	−173.3 (6)	C46—C47—C48—O3	173.8 (6)
C9—C7—C8—O1	−47.4 (8)	C49—C47—C48—O3	45.6 (9)
C11—C7—C8—O1	63.1 (8)	C51—C47—C48—O3	−65.6 (8)
C6—C7—C8—N1	8.3 (6)	C46—C47—C48—N9	−8.5 (6)
C9—C7—C8—N1	134.1 (5)	C49—C47—C48—N9	−136.7 (6)
C11—C7—C8—N1	−115.4 (5)	C51—C47—C48—N9	112.0 (5)
C12—N2—C9—C7	−157.0 (5)	C50—N10—C49—C47	36.7 (7)
C10—N2—C9—C7	−29.2 (7)	C52—N10—C49—C47	159.9 (6)
C6—C7—C9—N2	−127.9 (6)	C46—C47—C49—N10	121.7 (6)
C8—C7—C9—N2	114.0 (6)	C48—C47—C49—N10	−120.8 (6)
C11—C7—C9—N2	−0.6 (6)	C51—C47—C49—N10	−5.9 (7)
C12—N2—C10—C13	−62.9 (7)	C49—N10—C50—C53	−174.6 (5)
C9—N2—C10—C13	170.7 (5)	C52—N10—C50—C53	63.5 (6)
C12—N2—C10—C11	173.1 (5)	C49—N10—C50—C51	−50.8 (5)
C9—N2—C10—C11	46.7 (5)	C52—N10—C50—C51	−172.7 (5)
N2—C10—C11—C19	−168.5 (4)	N10—C50—C51—C60	−72.3 (5)
C13—C10—C11—C19	68.7 (6)	C53—C50—C51—C60	51.0 (7)
N2—C10—C11—C20	71.5 (5)	N10—C50—C51—C59	168.7 (4)
C13—C10—C11—C20	−51.2 (6)	C53—C50—C51—C59	−68.0 (6)
N2—C10—C11—C7	−45.5 (5)	N10—C50—C51—C47	44.7 (5)
C13—C10—C11—C7	−168.3 (4)	C53—C50—C51—C47	168.1 (5)
C6—C7—C11—C19	−82.2 (6)	C46—C47—C51—C60	−36.1 (6)
C8—C7—C11—C19	31.7 (6)	C49—C47—C51—C60	94.4 (5)
C9—C7—C11—C19	151.1 (5)	C48—C47—C51—C60	−147.0 (5)
C6—C7—C11—C20	36.0 (6)	C46—C47—C51—C59	83.9 (6)
C8—C7—C11—C20	149.9 (4)	C49—C47—C51—C59	−145.6 (5)
C9—C7—C11—C20	−90.8 (5)	C48—C47—C51—C59	−27.0 (6)
C6—C7—C11—C10	154.5 (5)	C46—C47—C51—C50	−154.0 (4)
C8—C7—C11—C10	−91.6 (5)	C49—C47—C51—C50	−23.5 (6)
C9—C7—C11—C10	27.8 (5)	C48—C47—C51—C50	95.1 (5)
N2—C10—C13—C14	−39.1 (7)	N10—C50—C53—C54	36.5 (8)
C11—C10—C13—C14	75.9 (7)	C51—C50—C53—C54	−77.7 (7)
N2—C10—C13—C18	140.2 (5)	N10—C50—C53—C58	−144.6 (6)
C11—C10—C13—C18	−104.7 (6)	C51—C50—C53—C58	101.1 (6)
C18—C13—C14—C15	1.9 (9)	C58—C53—C54—C55	−0.9 (10)
C10—C13—C14—C15	−178.8 (5)	C50—C53—C54—C55	177.9 (6)
C13—C14—C15—C16	−1.6 (10)	C53—C54—C55—C56	−0.8 (11)
C14—C15—C16—C17	0.3 (11)	C54—C55—C56—C57	1.1 (12)
C15—C16—C17—C18	0.9 (11)	C55—C56—C57—C58	0.4 (12)
C14—C13—C18—C17	−0.7 (9)	C54—C53—C58—C57	2.3 (9)
C10—C13—C18—C17	179.9 (5)	C50—C53—C58—C57	−176.6 (6)
C16—C17—C18—C13	−0.7 (10)	C56—C57—C58—C53	−2.1 (11)
C28—N5—C21—C26	−4.2 (6)	C68—N13—C61—C66	5.4 (7)
C28—N5—C21—C22	174.6 (5)	C68—N13—C61—C62	−172.8 (6)
C26—C21—C22—C23	1.8 (9)	C66—C61—C62—C63	−0.1 (9)
N5—C21—C22—C23	−176.9 (5)	N13—C61—C62—C63	177.9 (6)
C21—C22—C23—C24	0.5 (10)	C61—C62—C63—C64	−2.0 (10)
C22—C23—C24—C25	0.1 (11)	C62—C63—C64—C65	3.0 (11)
C23—C24—C25—C26	−3.0 (11)	C63—C64—C65—C66	−1.8 (10)
C22—C21—C26—C25	−4.6 (8)	C62—C61—C66—C65	1.3 (8)
N5—C21—C26—C25	174.2 (5)	N13—C61—C66—C65	−177.1 (5)
C22—C21—C26—C27	−179.7 (5)	C62—C61—C66—C67	178.4 (5)
N5—C21—C26—C27	−0.9 (6)	N13—C61—C66—C67	0.0 (6)
C24—C25—C26—C21	5.1 (9)	C64—C65—C66—C61	−0.3 (9)
C24—C25—C26—C27	178.9 (6)	C64—C65—C66—C67	−176.7 (6)
C21—C26—C27—C29	126.2 (5)	C61—C66—C67—C69	−127.7 (5)
C25—C26—C27—C29	−48.0 (8)	C65—C66—C67—C69	49.0 (8)
C21—C26—C27—C28	4.7 (5)	C61—C66—C67—C68	−4.5 (5)
C25—C26—C27—C28	−169.5 (6)	C65—C66—C67—C68	172.2 (6)
C21—C26—C27—C31	−112.7 (5)	C61—C66—C67—C71	112.8 (5)
C25—C26—C27—C31	73.1 (7)	C65—C66—C67—C71	−70.5 (7)
C21—N5—C28—O2	−173.5 (5)	C61—N13—C68—O4	174.1 (5)
C21—N5—C28—C27	7.2 (6)	C61—N13—C68—C67	−8.3 (6)
C26—C27—C28—O2	173.5 (5)	C66—C67—C68—O4	−174.7 (5)
C29—C27—C28—O2	48.3 (7)	C69—C67—C68—O4	−49.1 (7)
C31—C27—C28—O2	−64.5 (6)	C71—C67—C68—O4	63.6 (6)
C26—C27—C28—N5	−7.1 (5)	C66—C67—C68—N13	7.7 (5)
C29—C27—C28—N5	−132.3 (5)	C69—C67—C68—N13	133.4 (5)
C31—C27—C28—N5	114.9 (4)	C71—C67—C68—N13	−113.9 (5)
C30—N6—C29—C27	37.0 (6)	C70—N14—C69—C67	−38.3 (6)
C32—N6—C29—C27	162.6 (5)	C72—N14—C69—C67	−159.9 (5)
C26—C27—C29—N6	116.1 (5)	C66—C67—C69—N14	−115.8 (5)
C28—C27—C29—N6	−127.8 (5)	C68—C67—C69—N14	127.7 (5)
C31—C27—C29—N6	−11.4 (6)	C71—C67—C69—N14	10.7 (6)
C32—N6—C30—C33	66.4 (6)	C69—N14—C70—C73	169.6 (4)
C29—N6—C30—C33	−168.8 (4)	C72—N14—C70—C73	−68.8 (6)
C32—N6—C30—C31	−170.8 (5)	C69—N14—C70—C71	48.1 (5)
C29—N6—C30—C31	−46.1 (5)	C72—N14—C70—C71	169.7 (5)
N6—C30—C31—C40	158.4 (4)	N14—C70—C71—C79	81.6 (5)
C33—C30—C31—C40	−76.7 (6)	C73—C70—C71—C79	−41.4 (6)
N6—C30—C31—C39	−81.9 (5)	N14—C70—C71—C80	−160.4 (4)
C33—C30—C31—C39	43.0 (6)	C73—C70—C71—C80	76.6 (5)
N6—C30—C31—C27	37.7 (5)	N14—C70—C71—C67	−38.9 (5)
C33—C30—C31—C27	162.5 (4)	C73—C70—C71—C67	−161.8 (4)
C26—C27—C31—C40	96.6 (6)	C66—C67—C71—C79	24.9 (6)
C29—C27—C31—C40	−135.0 (5)	C69—C67—C71—C79	−102.6 (5)
C28—C27—C31—C40	−16.7 (6)	C68—C67—C71—C79	137.4 (5)
C26—C27—C31—C39	−24.7 (6)	C66—C67—C71—C80	−96.5 (6)
C29—C27—C31—C39	103.7 (5)	C69—C67—C71—C80	136.0 (5)
C28—C27—C31—C39	−138.0 (4)	C68—C67—C71—C80	16.0 (6)
C26—C27—C31—C30	−144.1 (4)	C66—C67—C71—C70	144.8 (4)
C29—C27—C31—C30	−15.7 (5)	C69—C67—C71—C70	17.3 (5)
C28—C27—C31—C30	102.6 (4)	C68—C67—C71—C70	−102.7 (4)
N6—C30—C33—C38	32.4 (7)	N14—C70—C73—C74	155.5 (5)
C31—C30—C33—C38	−84.0 (6)	C71—C70—C73—C74	−91.0 (6)
N6—C30—C33—C34	−150.3 (5)	N14—C70—C73—C78	−26.4 (7)
C31—C30—C33—C34	93.2 (6)	C71—C70—C73—C78	87.2 (6)
C38—C33—C34—C35	2.1 (10)	C78—C73—C74—C75	−1.9 (9)
C30—C33—C34—C35	−175.3 (6)	C70—C73—C74—C75	176.3 (5)
C33—C34—C35—C36	−3.8 (11)	C73—C74—C75—C76	−0.3 (11)
C34—C35—C36—C37	3.9 (12)	C74—C75—C76—C77	3.2 (11)
C35—C36—C37—C38	−2.0 (12)	C75—C76—C77—C78	−3.9 (11)
C34—C33—C38—C37	−0.1 (10)	C76—C77—C78—C73	1.8 (11)
C30—C33—C38—C37	177.0 (6)	C74—C73—C78—C77	1.1 (9)
C36—C37—C38—C33	0.1 (11)	C70—C73—C78—C77	−177.1 (6)

### Hydrogen-bond geometry (Å, °)

**Table:**

*D*—H···*A*	*D*—H	H···*A*	*D*···*A*	*D*—H···*A*
N1—H1···O2	0.86	2.08	2.867 (6)	153
N5—H5···N16	0.86	2.40	3.184 (8)	151
N9—H9···O4^i^	0.86	2.10	2.874 (6)	150
N13—H13···N8	0.86	2.33	3.120 (8)	152

Symmetry codes: (i) *x*+1/2, *y*−1/2, *z*.
